# Supplementary material for: Proteome Profiling of the Dura Mater in Patients with Moyamoya Angiopathy
Source: Int J Mol Sci. 2023 Jul 7;24(13):11194. doi: 10.3390/ijms241311194 (PMC10342562; doi:10.3390/ijms241311194)
Supplement: Supplementary file 1 [file ijms-24-11194-s001.zip › Supplemental Table S1_Carrozzini T et al.pdf]

Table S1. Clinical and demographical characterization of MMA patients collected for DM proteome analysis.

		MMA patient 1	MMA patient 2
**Personal details**	Ethnicity	Caucasian	Caucasian
	Age at recruitment	47 yo	44 yo
	Sex	Female	Female
**Index event**	Age occurence event	46	43
	CVD type	TIA	Ischemic stroke
	CVD lesion site	-	Bilateral Frontal-parietal
	CVD presentation	Bilateral	Bilateral
	First event	No	Yes
	Number and type other events	>2, TIA	-
	NIHSS	-	8
	Suzuki	3	5
	Modified Rankin scale	0	1
**Associated symptoms and signs**	Headache	No	No
	Seizures	No	Yes
	Mental Disorders	No	No
	Uncontrolled movements	No	Yes
	Retinal alterations	No	Yes
	Cognitive decline	No	Yes
**Medical therapy**	Antiaggregants	Yes	Yes
	Acetyl salicylic acid	No	Yes
	Clopidrogel	No	No
	Anticoagulants	No	No
	Statins	No	Yes
	CA-antagonists	No	No
	Antiepileptic	No	Yes
**Family History**	At least 1 family member with stroke	Yes, Mother	No
	At least 1 family member with headache	No	No
	At least 1 family member with dementia	No	No
	At least 1 family member with mental disorders	No	No
	At least 1 family member with epilepsy	No	No
	At least 1 family member with aneurysm	No	No
	At least 1 family member with dissection	No	No
	At least 1 family member with MMA	No	No
**Neurological examination**	Force disorder	Yes	Yes
	Sensory disorders	No	Yes
	Aphasia	No	Yes
	Visual field disturbance	No	No
	Neglect	No	No
	Disorder of vigilance	No	No
	Dysarthria	No	No

	Ataxia	No	No
**Risk factors**	Weight	90 Kg	85 Kg
	Height	178 cm	171 cm
	Hypertension	Yes	Yes
	Diabetes mellitus	No	No
	Dyslipidemia	No	No
	Ischemic heart disease	No	No
	Atrial fibrillation	No	No
	Peripheral vascular disease	No	No
	History od smoke	No	No
	Physical inactivity	Yes	Yes
	Therapy with extropogestinics	No	No
	Hyperhomocysteinemia	No	No
	Substance abuse	No	No
	Head trauma	No	No
	Others	spontaneous abortion, dyshtyroidism, translocation chr 1:15	mental retardation, dysmorphism, eclampsia, microduplication of chr 15q13.3, microdelation of chr 18q21.32
**Surgical treatment**	Revascularization procedure	Yes	Yes
	Direct	Yes	Yes
	Indirect	Yes	Yes
	Type	STA-MCA bypass; Encephalo-Duro-Myo-Synangiosis (left)	STA-MCA bypass; Encephalo-Duro-Arterio-Synangiosis (right)
	Procedural complications	Yes, sub-dural hematoma	No
	Additional revascularization procedure	No	Yes
	Direct	-	Yes
	Indirect	-	Yes
	Type	-	STA-MCA left
	Procedural complications	-	Yes, dysphagia
**Diagnostic workout**	Techniques	ECDTSA, TC, AngioRMN, RMN perfusion, RMN DWI, AngioTC, AngioRMN NOVA, Cerebral Angiography,	ECDTSA, ECDTC, TC, MRI, AngioRMN, RMN perfusion, RMN DWI, AngioTC, Angio RMN NOVA, Cerebral Angiography, DWI
	Electroencephalogram	Yes	Yes
	Epileptic attack	Yes (T sx)	No
	NPS test	-	Yes: attention, problem solving, working memory
**Neuroradiological features**	Leukoaraiosis	0	3
	Right carotid artery	Stenosis < 50%	Stenosis >50%
	Left carotid artery	Stenosis < 50%	Stenosis >50%
	Right middle cerebral artery (MCA)	Stenosis < 50%	Occlusion
	Left middle cerebral artery (MCA)	Stenosis < 50%	Stenosis >50%
	Right anterior cerebral artery (ACA)	No	Stenosis >50%
	Left anterior cerebral artery (ACA)	No	Stenosis >50%

Abnormal vascular network	Yes	Yes
Cerebral blood volume	Normal	Reduced
Cerebral blood flow	Reduced	Reduced
Low-flow site	Bilateral hemisphere	Bilateral
Mean Transit Time	Enhanced	Enhanced
Hypoperfusion	Yes	Yes
Hypoperfusion site	Bilateral watershed	Left > dx

Abbreviations: ACA, anterior cerebral artery; CVD, cerebrovascular disease; DWI, diffusion weighted imaging; ECDTSA, echocolordoppler of the supra-aortic trunks; MCA, middle cerebral artery; MMA, Moyamoya angiopathy; MRI, Magnetic Resonance Imaging; NIHSS, National Institutes of Health Stroke Scale; NPS, neuropsychiatric test; RMN, Nuclear Magnetic Resonance; STA, superficial temporal artery; TC, Computed Tomography; TIA, transient ischemic attack.
